# Green tea consumption and dementia risk in community-dwelling Japanese people aged 40–74 years: A 12-year cohort study

**DOI:** 10.1016/j.jnha.2025.100615

**Published:** 2025-06-24

**Authors:** Rikuto Kaise, Kaori Kitamura, Yumi Watanabe, Keiko Kabasawa, Akemi Takahashi, Toshiko Saito, Ryosaku Kobayashi, Rieko Oshiki, Osamu Yamazaki, Kei Watanabe, Ribeka Takachi, Shoichiro Tsugane, Kazutoshi Nakamura

**Affiliations:** a Niigata University School of Medicine, Niigata, Japan; b Division of Preventive Medicine, Niigata University Graduate School of Medical and Dental Sciences, Niigata, Japan; c Department of Health Promotion Medicine, Niigata University Graduate School of Medical and Dental Sciences, Niigata, Japan; d Department of Rehabilitation, Niigata University of Rehabilitation, Niigata, Japan; e Niigata Prefectural Government, Niigata, Japan; f Department of Orthopedic Surgery, Niigata University Medical and Dental Hospital, Niigata, Japan; g Department of Food Science and Nutrition, Nara Women’s University Graduate School of Humanities and Sciences, Nara, Japan; h International University of Health and Welfare Graduate School of Public Health, Tokyo, Japan

**Keywords:** Alzheimer’s disease, Coffee, Cohort study, Dementia, Green tea, Interaction

## Abstract

**Objective:**

Green tea, like coffee, has been suggested to protect against dementia, but supporting evidence is lacking. The present study aimed to determine independent associations of green tea consumption with, and interactions of green tea and coffee consumption on, dementia risk in middle-aged and older people.

**Methods:**

The present study is a 12-year follow-up of the Murakami cohort study on age-related diseases. Participants were 13,660 (men, 6,573 [48.1%]; mean age, 59.0 (SD = 9.3) years) community-dwelling residents aged 40–74 years. The baseline survey was conducted between 2011−2013. A self-administered questionnaire obtained information on predictors, including sex, age, marital status, education, occupation, body size, physical activity, smoking, alcohol, tea and coffee consumption, energy intake, and medical history. Green tea consumption was quantitatively determined with a validated questionnaire. Cases of incident dementia were identified using the long-term care insurance database.

**Results:**

Higher green tea consumption was associated with lower hazard ratios (HRs) for dementia (multivariable P for trend = 0.0178), with the highest quartile having a lower HR (adjusted HR = 0.75) than the lowest quartile. The adjusted HR for dementia by cup-based green tea consumption (1 cup = 150 mL) was 0.952 (95%CI:0.92−0.99), corresponding to a 4.8% reduction per 1 cup increase. High consumption of both green tea and coffee was not associated with low dementia risk (P for interaction = 0.0210).

**Conclusion:**

Higher consumption of green tea is independently associated with a lower risk of dementia. Although green tea was found to be beneficial, excessive consumption of both green tea and coffee is not recommended for the prevention of dementia.

## 1 Introduction

Tea is a beverage widely consumed worldwide. Polyphenols such as catechins in tea have antioxidant and anti-inflammatory properties [1] which are beneficial in obesity prevention and reduce the risk of cancer, heart disease, diabetes, and all-cause mortality [2].

Tea has various neuroprotective effects in addition to antioxidant and anti-inflammatory effects [1], and experimental and epidemiological studies have suggested an inverse association between tea consumption and cognitive decline and the incidence of dementia (especially Alzheimer’s disease) [1,3]. A recently published meta-analysis showed a linear association between tea intake and dementia risk, with risk decreasing with each additional cup per day (0.96, 95% Cl 0.94−0.99) [3].

Green tea contains more catechins than other teas, such as black tea; the catechin content of green tea has been reported to be about 4 times higher than that of black tea [4,5]. However, most of the studies conducted worldwide on the association between tea and dementia have used black tea, and not many studies have been conducted using green tea. Indeed, of the 10 publications that examined the relationship between tea consumption and dementia between 2001 and 2023, only three examined green tea (two used a qualitative assessment for green tea consumption) [3]. Therefore, well-designed studies on the effect of green tea on dementia risk would be informative.

We have been conducting a cohort study since 2011 on age-related diseases, including dementia, as an outcome [6]. Using this framework, we previously reported a clear inverse association between coffee consumption and dementia risk in the 8-year follow-up [7]. The 8-year follow-up study also examined the association between green tea consumption and dementia risk; however, the effects of green tea, as well as the interaction between green tea and coffee consumption, on dementia risk were unclear [7]. The purpose of this study is to determine the association of green tea consumption with, and interaction of green tea and coffee consumption on, dementia risk among middle-aged and older Japanese people through a 12-year follow-up of the Murakami cohort study.

## 2 Methods

### 2.1 Study design and participants

The present study is a 12-year follow-up of the Murakami cohort study. The Murakami cohort study invited all 34,082 residents aged 40–74 years living in Murakami City, Sekikawa Village, and Awashimaura Village in Niigata Prefecture to participate in the study. Of these, 14,364 people voluntarily participated in the baseline survey. Of the 14,364 people, the following were excluded: 23 individuals who were already certified as needing long-term care at baseline, and 486 individuals with missing data on body mass index (BMI), education, smoking, alcohol intake, coffee intake, energy intake, or physical activity level. In addition, individuals with outlier values for BMI (n = 52) and energy intake (n = 143), defined as values exceeding 3 standard deviations (SD) from the mean, were excluded from the analysis. The final analysis population for the present follow-up study was 13,660 people. Written informed consent was obtained from all participants. The protocol of this study was approved by the Ethics Committee of Niigata University (Nos. 1324 and 2018-0417).

### 2.2 Baseline survey

The baseline survey of the Murakami cohort study was conducted over the period 2011−2013. A self-administered questionnaire was used to obtain information on sex, age, marital status, education, occupation, height, weight, physical activity, smoking, alcohol consumption, energy intake, and medical history. Body mass index (BMI) was calculated by dividing weight (kg) by the square of height (m^2^). Self-reported height and weight were evaluated for validity using a sub-sample of the study population by comparing them with directly measured values. The results showed strong agreement, with Spearman’s correlation coefficients of 0.974 for height and 0.972 for weight in men, and 0.975 for height and 0.973 for weight in women (n = 1,752 men and 2,259 women; all P < 0.0001) [6]. Codes for demographics, smoking, and alcohol consumption are shown in Supplementary Table S1. Total physical activity levels in the last year were estimated by the METs index (MET-h/d), calculated as the intensity of each activity (Supplementary Table S1) multiplied by the number of hours per day. This method (JPHC-PAQ) was validated previously [8]. Energy intake was estimated with a validated, self-administered food frequency questionnaire [9]. For medical history, information on stroke, myocardial infarction, and diabetes mellitus was used.

For tea (green tea, black tea, and oolong tea) and coffee intake, we obtained information from a questionnaire on how much and how many beverages were consumed per day and calculated the number of drinks consumed in mL per day. Specifically, the options were “<1 cup per week,” “1−2 cups per week,” “3−4 cups per week,” “5−6 cups per week,” “1 cup per day,” “2−3 cups per day,” “4−6 cups per day,” “7−9 cups per day,” and 10 cups per day,” and total drinking volume was calculated assuming 200 mL for bottled tea, 250 mL for canned coffee, and 120 mL for tea and coffee other than canned or bottled. In Japan, brewed green tea and coffee are generally consumed hot, typically using smaller cups, whereas tea and coffee in cans or bottles are usually consumed cold and using larger cups. The cup volumes we used were based on a previous cohort study that used the same questionnaire [10]. Spearman’s energy-adjusted correlation coefficients between the present method and 12-day food record for green tea consumption were 0.66 for men and 0.80 for women [9]. Corresponding correlation coefficients for reproducibility for green tea were 0.65 for men and 0.76 for women [11]. Details of the baseline survey have been published elsewhere [6].

### 2.3 Follow-up

Cases of incident dementia were identified using the long-term care insurance (LTCI) database, a national system of social care for frail and elderly patients over 40 years of age. In the LTCI, physicians assess the degree of dementia and classify patients into six ranks ranging from no dementia (0) to severe dementia-related behavioral impairment and cognitive impairment requiring treatment (V). Those with rank II (moderate dementia-related behavioral disturbances and cognitive dysfunction with mild dependence) or higher are considered to have dementia. This criterion was reported to have high specificity (94%–97%) [12], and has been used in previous epidemiological studies. This method has been described in more detail in a previous study [13]. To calculate follow-up person-years, information on deaths and moving out of the study area was obtained based on the Basic Residential Registry Law and Family Registry Law.

### 2.4 Statistical methods

Medians and interquartile ranges were calculated for continuous variables and numbers (and percentages) for dummy variables. For BMI and energy intake, 52 and 143 cases, respectively, with log-transformed values exceeding ±3SD, were considered outliers and excluded from the statistical analysis. Hazard ratios (HRs) and P for trend values for dementia according to quartiles (Q) of green tea consumption were determined using a Cox proportional hazards model with the number of years of follow-up as the time variable. In the multivariate model, sex, age, marital status (dummy variable), education, occupation (dummy variable), log-transformed BMI, total physical activity, smoking, alcohol intake, coffee consumption, other tea (black tea and oolong tea) consumption, log-transformed energy intake, and medical history (stroke, myocardial infarction, diabetes) were covariates. We also created nine groups with three different green tea and coffee combinations (1, Q1; 2, Q2&3 [HRs of green tea Q2 and Q3 appeared similar]; and 3, Q4), and obtained multivariate-adjusted HRs for dementia using the same covariates previously stated, to assess the interaction of green tea and coffee consumption on dementia hazard. Finally, because dementia is a disease with a potentially long latency period and may affect tea drinking habits at baseline, we analyzed the association between green tea consumption and dementia risk in a subgroup that excluded dementia cases that developed within six years of the start of follow-up. Statistical analysis was performed using SAS statistical software (release 9.4, SAS Institute Inc.). P < 0.05 was considered statistically significant.

## 3 Results

The mean age of participants was 59.0 (SD = 9.3, N = 13,660) years, and the mean follow-up period was 11.5 (SD = 2.4) years. The number of men was 6,573 (48.1%). The numbers of new cases of dementia during the follow-up period were 12 (0.5%), 50 (1.3%), 284 (5.6%), and 336 (16.2%) for participants in their 40 s, 50 s, 60 s, and 70 s, respectively. Participant characteristics at baseline by green tea consumption quartile are shown in Table 1. Higher green tea consumption was positively associated with older age, total physical activity, energy intake, proportion of married individuals, and history of diabetes, and inversely associated with BMI, coffee consumption, and proportions of men, university graduates, manual job workers, smokers, and drinkers. Participant characteristics by sex are shown in Supplementary Table S2.

**Table 1:** Participant characteristics at baseline according to quartiles of green tea consumption.

	Quartiles of green tea consumption (mL/day)				P for trend
	Q1 (< 94)	Q2 (94-299)	Q3 (300-599)	Q4 (≥600)	
Number	3234	3265	3730	3431	
Age (years)	56 (48,63)	57 (49,63)	61 (54,68)	64 (59,69)	<0.0001
BMI (kg/m^2^)	22.9 (21.0,25.2)	23.1 (21.2,25.3)	22.9 (21.0,24.8)	22.8 (20.9,24.9)	<0.0001
Total physical activity (MET-h/d)	42.6 (37.1,53.0)	42.1 (36.6,51.8)	43.0 (37.1,53.1)	43.3 (37.8,53.7)	0.0315
Energy intake (kcal/d)	1778 (1392,2258)	1926 (1537,2421)	1929 (1558,2428)	2010 (1589,2521)	<0.0001
Green tea consumption (mL/d)	26 (0,60)	120 (120,163)	300 (300,343)	643 (600,960)	<0.0001
Black tea consumption (mL/d)	0 (0,0)	0 (0,26)	0 (0,0)	0 (0,0)	0.4935
Oolong tea consumption (mL/d)	0 (0,43)	0 (0,43)	0 (0,43)	0 (0,43)	0.1127
Coffee consumption (mL/d)	174 (54,354)	151 (60,300)	120 (26,300)	86 (0,245)	<0.0001
Men	1641 (50.7%)	1730 (53.0%)	1764 (47.3%)	1438 (41.9%)	<0.0001
Married	2533 (78.3%)	2592 (79.4%)	3116 (83.5%)	2821 (82.2%)	<0.0001
University graduates	195 (6.0%)	215 (6.6%)	237 (6.4%)	145 (4.2%)	0.0022
Manual job	778 (24.1%)	774 (23.7%)	808 (21.6%)	683 (19.9%)	<0.0001
Current smoker	820 (25.4%)	732 (22.4%)	640 (17.2%)	485 (14.1%)	<0.0001
Current drinker (alcohol)	1969 (60.9%)	2028 (62.1%)	2122 (56.9%)	1650 (48.1%)	<0.0001
History of stroke	69 (2.1%)	68 (2.1%)	70 (1.9%)	84 (2.4%)	0.4841
History of myocardial infarction	16 (0.5%)	21 (0.6%)	25 (0.7%)	24 (0.7%)	0.3118
History of diabetes	210 (6.5%)	239 (7.3%)	296 (7.9%)	312 (9.1%)	<0.0001

Data are presented as median with interquartile range or number.

MET: metabolic equivalent.

The number of and HRs for dementia cases according to green tea consumption quartiles are shown in Table 2. Higher green tea consumption quartiles were associated with lower hazards for dementia (multivariable P for trend = 0.0178), with the highest quartile having a lower HR (multivariable HR = 0.75) than the reference (the lowest quartile). The multivariable HR for dementia by 1 cup increase of green tea (1 cup of green tea was considered to be 150 mL) was 0.952 (95%CI: 0.92−0.99, P for trend = 0.0160). This value corresponds to a 4.8% reduction in dementia risk with a 1 cup increase.

**Table 2:** Incidence rates and hazard ratios (HRs) for dementia according to quartiles of green tea consumption.

	Quartiles of green tea consumption (mL/day)				P for trend
	Q1 (< 94)	Q2 (94-299)	Q3 (300-599)	Q4 (≥600)	
Number of participants	3234	3265	3730	3431	
Number of dementia cases	137	119	203	223	
Person-years (P-Y)	37169	38221	42883	39378	
Incidence rate (/1000P-Y)	3.69	3.11	4.73	5.66	
Unadjusted HR (95% CI)	1 (Ref)	0.84 (0.66,1.07)	1.29 (1.04,1.60)	1.55 (1.25,1.91)	<0.0001
Age-adjusted HR (95% CI)	1 (Ref)	0.81 (0.63,1.03)	0.82 (0.66,1.01)	0.74 (0.60,0.92)	0.0138
Multivariate-adjusted HR* (95% CI)	1 (Ref)	0.83 (0.65,1.06)	0.84 (0.67,1.05)	0.75 (0.60,0.94)	0.0178

* Adjusted for sex, age, marital status, education, occupation, body mass index, total physical activity levels, smoking, drinking, other tea consumption, coffee consumption, energy intake, and disease history.

The number of and HRs for dementia cases according to sex and age group (<65 vs. ≥65 years) are shown in Table 3. There was no significant trend in the association between green tea consumption and hazards for dementia (multivariable P for trend = 0.1776) in men. Higher green tea consumption quartiles tended to have lower hazards for dementia (multivariate P for trend = 0.0510), with the highest quartile having a lower HR (multivariable HR = 0.73, 95% CI: 0.53−0.998) than the reference in women. In the <65 years age group, the HR for the highest quartile (multivariable HR = 0.62, 95% CI: 0.39−0.99) was significantly lower than that of the reference group. In the ≥65 years age group, higher green tea consumption quartiles tended to have lower hazards for dementia (multivariable P for trend = 0.0723).

**Table 3:** Incidence rates and hazard ratios (HRs) for dementia according to quartiles of green tea consumption by sex and age groups.

	Quartiles of green tea consumption (mL/day)				P for trend
	Q1 (< 94)	Q2 (94-300)	Q3 (300-600)	Q4 (≥600)	
Men					
Number of participants	1641	1730	1763	1438	
Number of dementia cases	74	63	102	108	
Person-years (P-Y)	18409	20046	19803	16002	
Incidence rate (/1000P-Y)	4.02	3.14	5.15	6.75	
Unadjusted HR (95% CI)	1 (Ref)	0.77 (0.55,1.08)	1.29 (0.96,1.74)	1.70(1.26,2.28)	<0.0001
Age-adjusted HR (95% CI)	1 (Ref)	0.79 (0.56,1.10)	0.84 (0.62,1.13)	0.84(0.63,1.14)	0.4043
Multivariate-adjusted HR* (95% CI)	1 (Ref)	0.78 (0.55,1.10)	0.82 (0.61,1.12)	0.78(0.57,1.06)	0.1776
Women					
Number of participants	1593	1535	1967	1993	
Number of dementia cases	63	56	101	115	
Person-years (P-Y)	18760	18174	23080	23375	
Incidence rate (/1000P-Y)	3.36	3.08	4.38	4.92	
Unadjusted HR (95% CI)	1 (Ref)	0.92 (0.64,1.31)	1.31 (0.95,1.79)	1.47 (1.08,2.00)	0.0019
Age-adjusted HR (95% CI)	1 (Ref)	0.82 (0.57,1.17)	0.80 (0.58,1.10)	0.68 (0.50,0.92)	0.0156
Multivariate-adjusted HR** (95% CI)	1 (Ref)	0.87 (0.60,1.25)	0.88 (0.64,1.22)	0.73 (0.53,1.00)	0.0510
<65 years of age					
Number of participants	2568	2570	2473	1784	
Number of dementia cases	50	33	49	32	
Person-years (P-Y)	30127	30705	29272	21189	
Incidence rate (/1000P-Y)	1.66	1.07	1.67	1.51	
Unadjusted HR (95% CI)	1 (Ref)	0.65 (0.42,1.00)	1.01 (0.68,1.49)	0.91(0.58,1.42)	0.8994
Age-adjusted HR (95% CI)	1 (Ref)	0.63 (0.41,0.98)	0.78 (0.53,1.16)	0.57(0.36,0.89)	<0.0347
Multivariate-adjusted HR** (95% CI)	1 (Ref)	0.68 (0.43,1.05)	0.90 (0.60,1.35)	0.62 (0.39,0.99)	0.1225
≥65 years of age					
Number of participants	666	695	1258	1647	
Number of dementia cases	87	86	154	191	
Person-years (P-Y)	7042	7516	13623	18188	
Incidence rate (/1000P-Y)	12.35	11.44	11.30	10.50	
Unadjusted HR (95% CI)	1 (Ref)	0.92 (0.68,1.24)	0.91 (0.70,1.19	0.84(0.65,1.08)	0.1734
Age-adjusted HR (95% CI)	1 (Ref)	0.91 (0.67,1.22)	0.85 (0.65,1.10)	0.82(0.63,1.05)	0.1084
Multivariate-adjusted HR** (95% CI)	1 (Ref)	0.92 (0.68,1.24)	0.84 (0.64,1.10)	0.80 (0.61,1.04)	0.0723

* Adjusted for age, marital status, education, occupation, body mass index, total physical activity levels, smoking, drinking, coffee consumption, other tea consumption, and disease history.

** Adjusted for sex, age, marital status, education, occupation, body mass index, total physical activity levels, smoking, drinking, coffee consumption, other tea consumption, and disease history.

**Fig. 1:**
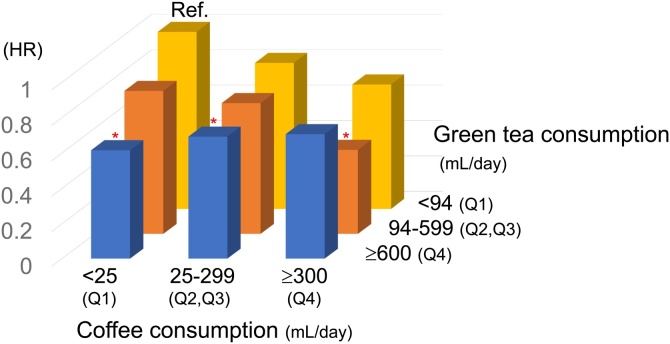
Adjusted hazard ratios (HRs) for dementia according to combined quartile-based green tea and coffee consumption, with the Q1 (<94-mL-green tea and <25-mL-coffee) group as the reference. Asterisks (*) denote HRs that are significantly lower or higher than the reference. Overall, HRs tended to decrease with increasing consumption of green tea and coffee, except for the Q4 (≥600 mL-green tea and ≥300 mL-coffee) groups. P for interaction of green tea and coffee consumption (both continuous variables) on dementia hazards was 0.0210.

We also created and assessed nine groups with different amounts of consumption of a combination of green tea and coffee (coffee consumption and dementia hazards shown in Supplementary Table S3), and obtained multivariate-adjusted HRs for dementia (Fig. 1). Overall, HRs tended to decrease with increasing consumption of green tea and coffee, except at Q4 (≥600 mL/d-green tea and ≥300 mL/d-coffee) levels. P for interaction of green tea and coffee consumption (both continuous variables) on dementia hazard was 0.0210.

The number of and HRs for dementia cases according to green tea consumption quartiles after excluding cases of dementia up to the sixth year of follow-up are shown in Supplementary Table S4. Higher green tea consumption quartiles were associated with lower hazards for dementia (multivariable P for trend = 0.0477), with the highest quartile having a significantly lower hazard (multivariable HR = 0.76) than the reference.

## 4 Discussion

The present study demonstrated a dose-dependent association between green tea consumption and risk reduction of dementia in middle-aged and older Japanese individuals, and high consumption of both green tea and coffee was not associated with a reduced risk of dementia.

The present study showed a 4.8% risk reduction for each 1 cup per day increase. A meta-analysis by Li et al. [3] showed that there is a linear association between tea consumption and risk of dementia, with a decreased risk of dementia (HR = 0.96, 95%CI: 0.93−0.99, for each 1 cup per day increase), i.e., a 4% decrease per 1 cup increase. The results of that meta-analysis study included tea in general. Although the definition of 1 cup may differ by study, the results of the present study, which examined only the effect of green tea, suggest that the effect of green tea on dementia risk reduction may be greater than that of other teas (e.g., black tea). Uchida et al. [14] conducted an RCT in which 1 cup of green tea (2 g green tea powder) per day was consumed, but found no significant change in cognitive function after one year, suggesting the potential difficulty of verifying the effect of consumption of green tea in small amounts.

Tomata et al. [15] conducted a cohort study of 31,694 adults (N = 13,645, age ≥65 years) followed for about six years and found that the risk of developing dementia decreased with a higher frequency of daily green tea consumption, with a hazard ratio of 0.73 for the group of five or more cups versus the group of less than one cup. This result is consistent with the results of the present study. However, the study by Tomata et al. [15] differed in that their analysis was based on the frequency of green tea cup consumption, while the present study estimated total green tea intake and frequency of intake of both bottled and brewed green tea.

Zhu et al. [16] noted that the effect of tea consumption on cognitive disorders was greater in Asians than in Caucasians. The reason for this is unclear, but a potential explanation is that most Asian studies used green tea consumption as an exposure, suggesting that green tea has a greater preventive effect than other teas, such as black tea. The effect of green tea on dementia is thought to be attributable to bioactive compounds found in green tea, such as catechins. Khokhar et al. [4] reported that the amount of catechins in green tea was 67.0 mg/g dry tea leaves, about 4.4 times that of black tea (15.4 mg/g), and Peterso et al. [5] reported that the amount of catechins in green tea was 156.7 mg/g dry tea leaves, about 4 times that of black tea (39.3 mg/g), suggesting that green tea contains a much higher amount of catechin than black tea.

Animal and in vitro studies have shown that various molecular mechanisms, including antioxidant effects, acetylcholinesterase (AChE) inhibition, reduction of amyloid-β accumulation and tau aggregation, and reduction of cerebrovascular disease risk by suppressing atherosclerosis, may contribute to the preventive effects of tea and bioactive compounds in tea on dementia [[16], [17], [18], [19], [20]]. Among these, epigallocatechin-3-gallate (EGCG), a major bioactive component of green tea catechins, has been proposed as a potential disease modulating compound for Alzheimer’s disease due to its diverse antioxidant and anti-inflammatory effects [21,22]. With regard to antioxidant effects, EGCG has been reported to be a more effective radical scavenger than vitamins E and C [16], and may prevent brain inflammation and neuronal damage [1]. EGCG decreases AChE activity and increases cholinergic communication, which could reduce cognitive deficits [17]. EGCG is also reported to reduce amyloid-β accumulation [18], and to prevent tau aggregation to rescue tau toxicity [19]. Furthermore, EGCG may suppress atherosclerosis and decrease cerebrovascular disease risk [20], which could lead to a reduction in vascular dementia. Recently, hydroxycinnamoylated catechins (HCCs) have been reported to be neuroprotective and have antioxidant effects [23]. Extracts of Longjing 43 green tea, which has high HCC content, and HCCs extracted from tea delayed the onset of paralysis and extended the lifespan of Alzheimer’s disease model nematodes [24]. Green tea also contains caffeine (20 mg/100 mL), which has neuroprotective effects [25] that may contribute to the prevention of dementia.

The present study found that consumption of a combination of green tea and coffee was not associated with a lower risk of dementia at Q4 (≥600 mL/d-green tea and ≥300 mL/d-coffee) levels. Since the benefits of tea and coffee are attributed to polyphenols and/or caffeine [3], this finding may be explained by an overdose of these components when both drinks are consumed in large amounts. With regard to caffeine intake, green tea 600 mL (caffeine content of green tea, 20 mg/100 mL [7]) and coffee 300 mL (caffeine content of coffee, 60 mg/100 mL [7]) contain 300 mg of caffeine, which may be harmful at such high levels relative to the current daily caffeine intake limit of 400 mg/d [26]. Moreover, the major polyphenols in green tea and coffee differ, so their interactions may also have an effect. Collectively, high intake of both coffee and green tea appears to have little benefit.

A strength of the present study is that it is a large, long-term cohort study using a validated green tea consumption assessment method. However, there are also some limitations worth noting. First, we did not assess the cognitive status of participants at baseline. Individuals with low cognitive function may consume less green tea, and this may have led to reverse causation between green tea consumption and dementia and overestimated the strength of this association. This is a major limitation of the present study. Second, lifestyle information, including green tea intake, was obtained by self-report, which may have led to misclassification. Third, this study obtained dementia cases from the LTCI database. This method has a high specificity (94%–97%) but not a high sensitivity (73%) [12], which may have resulted in patient selection bias. Finally, the pathotype of dementia was not investigated. However, it has been reported that about two-thirds of dementia cases are the Alzheimer’s type [27]. Therefore, the present results may reflect the risk of Alzheimer’s disease.

## 5 Conclusions

Higher consumption of green tea is associated with a lower risk of dementia among middle-aged and older people living in Japan, and there is an interaction of green tea and coffee consumption on dementia risk. Although a beneficial effect of green tea was demonstrated, excessive consumption of both green tea and coffee is not recommended for the prevention of dementia. Future studies that investigate the upper limit of green tea consumption and its impact on dementia prevention are warranted.

## CRediT authorship contribution statement

Study conception and design: RKa and KN; acquisition of data: KKi, AT, TS, RKo, RO, OY, RT, and KN; analysis and interpretation of data: RKa and KN; drafting of the manuscript: RKa; critical addition of important intellectual content to the manuscript: YW, KKa, RT, KW, and ST; revising of the manuscript: YW and KN. All authors reviewed and approved the final version.

## Ethics approval and consent to participate

The protocol of this study was approved by the Ethics Committee of Niigata University (Nos. 1324 and 2018-0417). Written informed consent was obtained from all participants.

## Declaration of Generative AI and AI-assisted technologies in the writing process

AI was not used in the writing process.

## Funding

This work was supported by JSPS KAKENHI Grant Numbers JP23249035 and JP15H04782, and the National Cancer Center Research and Development Fund [23-A31(toku) (since 2010)]. The funders had no role in the study design, data collection and analysis, decision to publish, or preparation of the manuscript.

## Data availability

We cannot provide individual data because study participants did not consent to have their data revealed to anyone outside the research group. However, the minimal dataset may be available upon ethical approval by the Ethics Committee of Niigata University.

## Declaration of competing interest

The authors have no competing interests.

## References

[1] Mandel S.A., Amit T., Weinreb O., Reznichenko L., Youdim M.B.H. (2008). Simultaneous manipulation of multiple brain targets by green tea catechins: a potential neuroprotective strategy for Alzheimer and Parkinson diseases. CNS Neurosci Ther.

[2] Shin S., Lee J.E., Loftfield E., Shu X.O., Abe S.K., Rahman M.S. (2022). Coffee and tea consumption and mortality from all causes, cardiovascular disease and cancer: a pooled analysis of prospective studies from the Asia Cohort Consortium. Int J Epidemiol.

[3] Li F., Liu X., Jiang B., Li X., Wang Y., Chen X. (2024). Tea, coffee, and caffeine intake and risk of dementia and Alzheimer’s disease: a systematic review and meta-analysis of cohort studies. Food Funct.

[4] Khokhar S., Magnusdottir S.G.M. (2002). Total phenol, catechin, and caffeine contents of teas commonly consumed in the United kingdom. J Agric Food Chem.

[5] Peterson J., Dwyer J., Bhagwat S., Haytowitz D., Holden J., Eldridge A.L. (2005). Major flavonoids in dry tea. J Food Compost Anal.

[6] Nakamura K., Takachi R., Kitamura K., Saito T., Kobayashi R., Oshiki R. (2018). The Murakami Cohort Study of vitamin D for the prevention of musculoskeletal and other age-related diseases: a Study protocol. Environ Health Prev Med.

[7] Matsushita N., Nakanishi Y., Watanabe Y., Kitamura K., Kabasawa K., Takahashi A. (2021). Association of coffee, green tea, and caffeine with the risk of dementia in older Japanese people. J Am Geriatr Soc.

[8] Kikuchi H., Inoue S., Odagiri Y., Ihira H., Inoue M., Sawada N. (2020). Intensity-specific validity and reliability of the Japan Public Health Center-based prospective study - physical activity questionnaire. Prev Med Rep.

[9] Yokoyama Y., Takachi R., Ishihara J., Ishii Y., Sasazuki S., Sawada N. (2016). Validity of short and long self-administered food frequency questionnaires in ranking dietary intake in middle-aged and elderly Japanese in the Japan Public Health Center-Based Prospective Study for the Next Generation (JPHC-Next) protocol area. J Epidemiol.

[10] Narita Z., Hidese S., Kanehara R., Tachimori H., Hori H., Kim Y. (2024). Association of sugary drinks, carbonated beverages, vegetable and fruit juices, sweetened and black coffee, and green tea with subsequent depression: a five-year cohort study. Clin Nutr.

[11] Maruyama K., Ikeda A., Ishihara J., Takachi R., Sawada N., Shimazu T. (2019). Food frequency questionnaire reproducibility for middle-aged and elderly Japanese. Asia Pac J Clin Nutr.

[12] Noda H., Yamagishi K., Ikeda A., Asada T., Iso H. (2018). Identification of dementia using standard clinical assessments by primary care physicians in Japan. Geriatr Gerontol Int.

[13] Nakamura K., Watanabe Y., Kitamura K., Kabasawa K., Someya T. (2019). Psychological distress as a risk factor for dementia after the 2004 Niigata–Chuetsu earthquake in Japan. J Affect Disord.

[14] Uchida K., Meno K., Korenaga T., Liu S., Suzuki H., Baba Y. (2024). Effect of matcha green tea on cognitive functions and sleep quality in older adults with cognitive decline: a randomized controlled study over 12 months. PLoS One.

[15] Tomata Y., Sugiyama K., Kaiho Y., Honkura K., Watanabe T., Zhang S. (2016). Green tea consumption and the risk of incident dementia in elderly Japanese: the Ohsaki Cohort 2006 Study. Am J Geriatr Psychiatry.

[16] Nanjo F., Goto K., Seto R., Suzuki M., Sakai M., Hara Y. (1996). Scavenging effects of tea catechins and their derivatives on 1,1- diphenyl-2-picrylhydrazyl radical. Free Radic Biol Med.

[17] Biasibetti R., Tramontina A.C., Costa A.P., Dutra M.F., Quincozes-Santos A., Nardin P. (2013). Green tea (-)epigallocatechin-3-gallate reverses oxidative stress and reduces acetylcholinesterase activity in a streptozotocin-induced model of dementia. Behav Brain Res.

[18] Secker C., Motzny A.Y., Kostova S., Buntru A., Helmecke L., Reus L. (2023). The polyphenol EGCG directly targets intracellular amyloid-β aggregates and promotes their lysosomal degradation. J Neurochem.

[19] Wobst H.J., Sharma A., Diamond M.I., Wanker E.E., Bieschke J. (2015). The green tea polyphenol (-)-epigallocatechin gallate prevents the aggregation of tau protein into toxic oligomers at substoichiometric ratios. FEBS Lett.

[20] Chyu K.Y., Babbidge S.M., Zhao X., Dandillaya R., Rietveld A.G., Yano J. (2004). Differential effects of green tea-derived catechin on developing versus established atherosclerosis in apolipoprotein E-null mice. Circulation.

[21] Valverde-Salazar V., Ruiz-Gabarre D., García-Escudero V. (2023). Alzheimer’s disease and green tea: epigallocatechin-3-gallate as a modulator of inflammation and oxidative stress. Antioxidants..

[22] Li S., Wang Z., Liu G., Chen M. (2024). Neurodegenerative diseases and catechins: (-)-epigallocatechin-3-gallate is a modulator of chronic neuroinflammation and oxidative stress. Front Nutr..

[23] Wang W., Zhang P., Liu X.H., Ke J.P., Zhuang J.H., Ho C.T. (2021). Identification and quantification of hydroxycinnamoylated catechins in tea by targeted UPLC-MS using synthesized standards and their potential use in discrimination of tea varieties. LWT.

[24] Yang Y., Ke J.P., Yang Z., Chen C.H., Li J.Y., Yu J.Y. (2024). Green tea hydroxycinnamoylated catechins extend lifespan and attenuate Aβ-induced paralysis of Caenorhabditis elegans via anti-oxidation and peptides dis-aggregation. Ind Crop Prod.

[25] Kolahdouzan M., Hamadeh M.J. (2017). The neuroprotective effects of caffeine in neurodegenerative diseases. CNS Neurosci Ther.

[26] Van Dam R.M., Hu F.B., Willett W.C. (2020). Coffee, caffeine, and health. N Engl J Med.

[27] Ohara T., Hata J., Yoshida D., Mukai N., Nagata M., Iwaki T. (2017). Trends in dementia prevalence, incidence, and survival rate in a Japanese community. Neurology.

