# Supplementary material for: Green tea consumption and dementia risk in community-dwelling Japanese people aged 40–74 years: A 12-year cohort study
Source: J Nutr Health Aging. 2025 Jun 24;29(8):100615. doi: 10.1016/j.jnha.2025.100615 (PMC12402371; doi:10.1016/j.jnha.2025.100615)
Supplement: Supplementary file 1 [file mmc1.docx]

Supplementary Table S1. Codes for demographic variables, smoking, and drinking, and the intensity of each physical activity (PA)

Variables

Marital status (a) married, (b) never married, and (c) divorced, separated, or bereaved

Education level (1) junior high school (9 years of education), (2) high school (12 years), (3) junior or

vocational college (14 years), and (4) university or graduate school (≥16 years)

Occupation (a) office work and sales/service work, (b) professional/management, (c) manual (security,

farming/forestry/fishery, transportation, and labor services), and (d) no job/others

Smoking (1) non-smoker, (2) past smoker, (3) 1–20 cigarettes/day, and (4) ≥20 cigarettes/day

Drinking (1) non- or rare drinker, (2) 1–149 g ethanol/week, (3) 150–299 g ethanol/week,

(4) 300–449 g ethanol/week, and (5) ≥450 g ethanol/week

Intensity of PA sitting (1.3 METs), standing (2.0 METs), walking (3.0 METs), strenuous work (6.0 METs),

walking slowly (2.8 METs), walking quickly (4.0 METs), light to moderate exercise

(3.0 METs), strenuous exercise (6.0 METs), sleep (0.9 METs), and other activities

(1.3 METs)

Supplementary Table S2. Participant characteristics at baseline according to quartiles of green tea consumption by sex.

Quartiles of green tea consumption (mL/day) P for trend

Q1 (< 94) Q2 (94-300) Q3 (300-600) Q4 (≥600)

Men

Number 1641 1730 1764 1438

Age (years) 57 (49,63) 57 (50,64) 62 (54,68) 65 (59,70) <0.0001

BMI (kg/m^2^) 23.5 (21.5,25.6) 23.6 (21.7,25.7) 23.3 (21.6,25.2) 23.3 (21.5,25.3) 0.0002

Total physical activity (MET-h/d) 43.7 (37.0,55.6) 43.0 (36.2,53.6) 43.5 (37.1,55.1) 44.8 (38.2,56.9) 0.0339

Energy intake (kcal/d) 1957 (1548,2418) 2110 (1699,2589) 2119 (1721,2598) 2203(1782,2733) <0.0001

Green tea consumption (mL/d) 26 (0,60) 126 (120,163) 320 (300,343) 643 (600,1002) <0.0001

Black tea consumption (mL/d) 0 (0,0) 0 (0,0) 0 (0,0) 0 (0,0) 0.3891

Oolong tea consumption (mL/d) 0 (0,43) 0 (0,43) 0 (0,43) 0 (0,43) 0.0704

Coffee consumption (mL/d) 180 (54,354) 174 (60,326) 120 (26,300) 79 (0,245) 0.1698

Married 1274 (77.6%) 1358 (78.5%) 1493 (84.7%) 1247 (86.7%) <0.0001

University graduates 144 (8.8%) 174 (10.1%) 178 (10.1%) 102 (7.1%) 0.2031

Manual job 501 (30.5%) 529 (30.6%) 511 (29.0%) 453 (31.5%) 0.8965

Current smoker 632 (38.5%) 612 (35.4%) 541 (307%) 402 (28.0%) <0.0001

Current drinker (alcohol) 1337 (81.5%) 1446 (83.6%) 1461 (82.9%) 1078 (75.0%) <0.0001

History of stroke 46 (2.8%) 46 (2.7%) 43 (2.4%) 49 (3.4%) 0.4456

History of myocardial infarction 14 (0.9%) 21 (1.2%) 20 (1.1%) 21 (1.5%) 0.1631

History of diabetes 150 (9.1%) 164 (9.5%) 196 (11.1%) 185 (12.9%) 0.0002

Women

Number 1593 1535 1967 1993

Age (years) 56 (48,63) 57 (48,63) 60 (53,67) 64 (58,69) <0.0001

BMI (kg/m^2^) 22.3 (20.2,24.7) 22.5 (20.7,24.7) 22.3 (20.4,24.5) 22.4 (20.5,24.7) 0.2478

Total physical activity (MET-h/d) 41.7 (37.1,51.3) 41.2 (36.8,50.1) 42.5 (37.2,51.3) 42.6 (37.7,51.3) 0.0663

Energy intake (kcal/d) 1632 (1297,2046) 1737 (1387,2181) 1785 (1462,2241) 1875 (1485,2356) <0.0001

Green tea consumption (mL/d) 26 (0,60) 120 (103,163) 300 (300,343) 643 (600,960) <0.0001

Black tea consumption (mL/d) 0 (0,26) 0 (0,26) 0 (0,26) 0 (0,26) 0.0138

Oolong tea consumption (mL/d) 0 (0,43) 0 (0,43) 0 (0,43) 0 (0,26) 0.0005

Coffee consumption (mL/d) 174 (60,326) 120 (54,300) 120 (54,300) 86 (26,245) 0.0768

Married 1259 (79.0%) 1234 (80.4%) 1623 (82.5%) 1574 (79.0%) 0.8649

University graduates 51 (3.2%) 41 (2.7%) 59 (3.0%) 43 (2.2%) 0.0983

Manual job 277 (17.4%) 245 (16.0%) 296 (15.1%) 230 (11.5%) <0.0001

Current smoker 188 (11.8%) 120 (7.8%) 99 (5.0%) 83 (4.2%) <0.0001

Current drinker (alcohol) 632 (39.7%) 582 (37.9%) 660 (33.6%) 572 (28.7%) <0.0001

History of stroke 23 (1.4%) 22 (1.4%) 27 (1.4%) 35 (1.8%) 0.4411

History of myocardial infarction 2 (0.1%) 0 (0.0%) 5 (0.3%) 3 (0.2%) 0.4507

History of diabetes 60 (3.8%) 75 (4.9%) 100 (5.1%) 127 (6.4%) 0.0006

Data are presented as median with interquartile range or number.

MET: metabolic equivalent

Supplementary Table S3. Incidence rates and hazard ratios (HRs) for dementia according to quartiles of coffee consumption.

Quartiles of coffee consumption P for trend

Q1 (<26) Q2 (26-119) Q3 (120-299) Q4 (≥300)

Number of participants 275 145 143 119

Number of dementia cases 3621 2255 3372 4412

Person-years (P-Y) 40646 25854 39254 51895

Incidence rate (/1000P-Y) 6.77 5.61 3.64 2.29

Unadjusted HR (95% CI) 1 (Ref) 0.82 (1.00-0.82) 0.53 (0.65-0.53) 0.33 (0.41-0.33) <0.0001

Age-adjusted HR (95% CI) 1 (Ref) 1.05 (1.28-1.05) 0.87 (1.07-0.87) 0.79 (0.98-0.79) 0.0208

Multivariate-adjusted HR^*^ (95% CI) 1 (Ref) 1.06 (1.30-1.06) 0.86 (1.05-0.86) 0.73 (0.91-0.73) 0.0038

^*^Adjusted for sex, age, marital status, education, occupation, body mass index, total physical activity levels, smoking, drinking, green tea consumption, other tea consumption, energy intake, and disease history

Supplementary Table S4. Incidence rates and hazard ratios (HRs) for dementia according to quartiles of green tea consumption after excluding dementia cases occurring within the first six years of follow-up

Quartiles of green tea consumption (mL/day) P for trend

Q1 (<94) Q2 (94-300) Q3 (300-600) Q4 (≥600)

Number of participants 3202 3236 3677 3382

Number of dementia cases 105 90 149 174

Person-years (P-Y) 37071 38128 42690 39182

Incidence rate (/1000P-Y) 2.83 2.36 3.49 4.44

Unadjusted HR (95% CI) 1 (Ref) 0.83 (0.62,1.10) 1.24 (0.97,1.59) 1.58 (1.24,2.02) <0.0001

Age-adjusted HR (95% CI) 1 (Ref) 0.80 (0.60,1.05) 0.78 (0.61,1.00) 0.76 (0.59,0.97) 0.0457

Multivariate-adjusted HR^*^ (95% CI) 1 (Ref) 0.81 (0.61,1.08) 0.80 (0.62,1.03) 0.76 (0.59,0.98) 0.0477

^*^Adjusted for sex, age, marital status, education, occupation, BMI, total physical activity levels, smoking, drinking, coffee consumption, other tea consumption, energy intake, and disease history
